# The exenatide analogue AC3174 attenuates hypertension, insulin resistance, and renal dysfunction in Dahl salt-sensitive rats

**DOI:** 10.1186/1475-2840-9-32

**Published:** 2010-08-03

**Authors:** Que Liu, Lisa Adams, Anatoly Broyde, Rayne Fernandez, Alain D Baron, David G Parkes

**Affiliations:** 1 Amylin Pharmaceuticals, Inc., San Diego, CA, USA

## Abstract

**Background:**

Activation of glucagon-like peptide-1 (GLP-1) receptors improves insulin sensitivity and induces vasodilatation and diuresis. AC3174 is a peptide analogue with pharmacologic properties similar to the GLP-1 receptor agonist, exenatide. Hypothetically, chronic AC3174 treatment could attenuate salt-induced hypertension, cardiac morbidity, insulin resistance, and renal dysfunction in Dahl salt-sensitive (DSS) rats.

**Methods:**

DSS rats were fed low salt (LS, 0.3% NaCl) or high salt (HS, 8% NaCl) diets. HS rats were treated with vehicle, AC3174 (1.7 pmol/kg/min), or GLP-1 (25 pmol/kg/min) for 4 weeks via subcutaneous infusion. Other HS rats received captopril (150 mg/kg/day) or AC3174 plus captopril.

**Results:**

HS rat survival was improved by all treatments except GLP-1. Systolic blood pressure (SBP) was lower in LS rats and in GLP-1, AC3174, captopril, or AC3174 plus captopril HS rats than in vehicle HS rats (p < 0.05). AC3174 plus captopril attenuated the deleterious effects of high salt on posterior wall thickness, LV mass, and the ratio of LV mass to body weight (P ≤ 0.05). In contrast, GLP-1 had no effect on these cardiovascular parameters. All treatments reduced LV wall stress. GLP-1, AC3174, captopril, or AC3174 plus captopril normalized fasting insulin and HOMA-IR (P ≤ 0.05). AC3174, captopril, or AC3174 plus captopril improved renal function (P ≤ 0.05). Renal morphology in HS rats was associated with extensive sclerosis. Monotherapy with AC3174, captopril, or GLP-1 attenuated renal damage. However, AC3174 plus captopril produced the most effective improvement.

**Conclusions:**

Thus, AC3174 had antihypertensive, cardioprotective, insulin-sensitizing, and renoprotective effects in the DSS hypertensive rat model. Furthermore, AC3174 improved animal survival, an effect not observed with GLP-1.

## Background

Cardiovascular disease is the number one cause of death in the United States.^1 ^In 2003, ~65 million adults had diagnosed hypertension, a key risk factor for cardiovascular disease and kidney failure [1,2]. Congestive heart failure with left ventricular (LV) dysfunction is often found in patients with hypertension [2-6]. In fact, hypertension is the strongest risk factor for heart failure. The transition from LV wall hypertrophy compensatory for abnormal wall stress to overt heart failure has long been recognized, but the underlying mechanisms remain poorly understood. However, it is known that during this transition insulin resistance develops, cardiac glucose uptake down-regulates, angiotensin-converting enzyme (ACE) levels increase, and the renin-angiotensin aldosterone system (RAAS) becomes hyperactivated [2,4,6].

Heart failure and diabetes are intrinsically linked [7]. Diabetes is a risk factor for coronary atherosclerosis leading to myocardial ischemia and infarction. Diabetes also causes cardiomyopathy independent of coronary atherosclerosis. Clinical presentation involves diastolic dysfunction characterized by abnormal LV relaxation, reduced systolic function and increased myocardial reflectivity, and elevated insulin resistance.

Hypertension and diabetes are the two leading causes of chronic kidney disease [2,8]. Drugs that improve glucose uptake and glucose oxidation have cardioprotective effects and can attenuate subsequent renal disease [2]. Glucagon-like peptide-1 (GLP-1) is an incretin hormone with insulinotropic properties that regulates glucose metabolism [9]. GLP-1 receptor agonists can attenuate insulin resistance and improve glycemic control in patients with type 2 diabetes. Intravenous infusion of GLP-1 in patients with acute myocardial infarction for 72 hours after successful angioplasty reportedly improved cardiac function [10]. Further, in pigs [11] and dogs [12] GLP-1 improved myocardial glucose-uptake and metabolism. In Dahl salt-sensitive (DSS) hypertensive rats, GLP-1 attenuated the development of hypertension and cardiac remodeling, reduced renal proteinuria and albuminuria, and improved functionality in both organs [13].

Exenatide is a peptide incretin mimetic that shares many glucoregulatory properties with GLP-1 [14-16]. *In vitro*, exenatide binds to and activates the known mammalian GLP-1 receptor. *In vivo*, exenatide enhances glucose-dependent insulin secretion, enhances glucose-dependent suppression of inappropriately high glucagon secretion, slows gastric emptying, and reduces food intake. In diabetes models, exenatide can promote β-cell proliferation and islet neogenesis from precursor cells [14-17]. In diabetes patients, 30 weeks of exenatide reduced mean HbA_1c _~1% with weight loss, effects that were sustained out to 3 years in open-label extensions [15,16]. Exenatide and GLP-1 improved hypertension, insulin sensitivity, vasodilatation, and renal diuresis in animal studies [13,17-20]. In both healthy and insulin-resistant obese men, GLP-1 similarly induced natriuresis [21]. In an open-label, 82-week study, exenatide reduced mean diastolic BP and improved lipid profiles [22]. In a 24-week clinical trial, exenatide reduced mean systolic and diastolic BP in contrast to non-significant BP changes in the placebo arm [23]. The BP effects of exenatide treatment lasting at least 6 months was also examined in pooled data from 6 trials including 2,171 subjects [24]. Exenatide was associated with significantly decreased systolic BP compared with placebo or insulin in patients with elevated BP at baseline, with the greatest effects observed in subjects with baseline systolic BP ≥ 130 mmHg.

The Dahl salt-sensitive (DSS) rat is a well established model for salt-induced hypertension and renal failure. DSS rats fed a high-salt diet (8% NaCl) develop diastolic heart dysfunction characterized by LV hypertrophy and increased LV myocardial thickness and stiffening [25-28], with elevated plasma insulin and triglyceride concentrations coupled with impaired insulin-stimulated glucose transport into cardiac muscle [25,29]. After 7 weeks, kidneys are characterized by decreased function, increased proteinuria, glomerulosclerosis, increased adrenomedullin and atrial natriuretic peptide concentrations compared with salt-resistant rats [30]. By one year of age, DSS rats on low-salt diet develop glomerulosclerosis and tubulointerstitial fibrosis similar to the age-related renal changes observed in humans [31].

The purpose of the present study was to determine whether hypertension, insulin resistance, and renal dysfunction in DSS rats fed a high-salt diet could be attenuated by the exenatide analogue, AC3174 [32], alone or in combination with the ACE inhibitor captopril. The efficacy of AC3174 was also compared to native GLP-1 administered at a dose 12X higher than the AC3174 dose (on a μg/kg/d basis). Compared with exenatide, AC3174 has a single amino acid substitution (leucine for methionine at position 14) to eliminate oxidation at this amino acid and potentially improve peptide stability [32]. *In vitro *AC3174 and exenatide have equivalent potency for displacement of GLP-1 from its receptor and for receptor activation. *In vivo*, AC3174 had efficacy similar to exenatide for decreasing ambient and postprandial plasma glucose.

## Methods

Male 7-week old Dahl salt-sensitive (DSS) rats [33] (209 ± 3 g; Harlan Sprague Dawley, Madison, WI) were fed low salt (0.3% NaCl) or high salt (8% NaCl) chow. High salt rats were treated for 4 weeks with vehicle or AC3174 [32] (1.7 pmol/kg/min [10 μg/kg/day]) via subcutaneous infusion (Alzet^® ^pump), with captopril in drinking water at a dose of 150 mg/kg/day, with AC3174 plus captopril, or with GLP-1 at a dose of 25 pmol/kg/min (120 μg/kg/day). AC3174 and GLP-1 doses were selected to provide equivalent plasma exposure [34]. For implantation of the Alzet osmotic mini-pumps, rats were anesthetized with ketamine (50-90 mg/kg) plus xylazine (5-10 mg/kg), a small subcutaneous pocket was created at the neck nape, the pump was inserted into the pocket, and the incision closed with wound clips. All procedures were approved by an Institutional Animal Care and Use Committee

Systolic blood pressure (BP) was measured by tail cuff (AD Instruments, Colorado Springs, CO) or telemetry via an implanted transmitter (DSI transducer, DSI, St. Paul, MN) placed into the right femoral artery or abdominal aorta, as instructed by the manufacturer. Rats were conscious and freely moving in their cages while blood pressure measurements were collected by telemetry. Cardiac function was assessed by transthoracic echocardiography using a HP Sonos 5500 ultrasound system (Davis Medical, San Diego, CA). Body temperature was maintained by a warm water circulating heating blanket. After completion of the observation period, animals were euthanized by overdosing with isoflurane and exsanguination was performed by cardiac puncture.

The homoeostasis model assessment-insulin resistance (HOMA-IR) index was calculated from fasting plasma glucose and insulin concentrations according to the equation (insulin*glucose)/22.5. A bolus intraperitoneal injection of 2 g glucose/kg body weight was administered for the glucose tolerance test at week 5. Rats were fasted overnight for at least 6 hours for fasting measurements. Blood glucose samples were obtained via tail vein.

Renal function was evaluated by measuring serum and urinary creatinine concentrations and by calculating renal creatinine clearance rates according to the equation: Clearance rate (ml/min) = (urine creatinine concentration*urine volume)/serum creatinine concentration/time period.

Tissue samples were fixed via intracardiac perfusion with 10% buffered formalin and paraffin-embedded overnight in a Miles VIP Tissue Processor. Serial 8 micron thick sections were cut with a Leitz microtome, stained with a routine Harris hematoxylin and eosin stain or trichrome blue, photographed using an Olympus BH2 Fluorescent microscope with a SpotRT digital camera (Olympus, Temecula, CA), and analyzed using Image-Pro Plus 4.1 software (DataCell Ltd, North Chelmsford, MA).

Students' t-tests, one-way ANOVA followed by Bonferroni multiple comparison tests, or two-way ANOVA followed by Bonferroni multiple comparison tests were used to test treatment group differences at defined time points. Repeated measures analyses and Dunnett's tests were used to compare treatment groups to the high salt group over time. Animal survival was evaluated using Kaplan-Meier survival curves. Data are presented as mean ± SEM.

## Results

**Figure 1:**
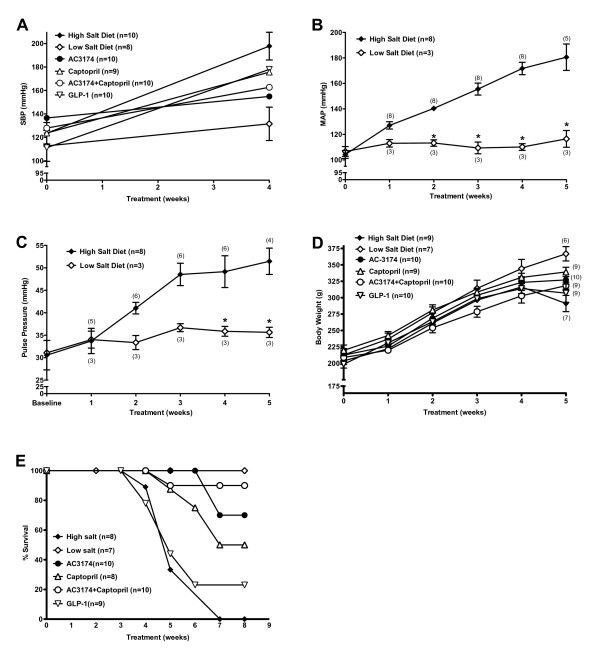
**Development of hypertension in the DSS rat over 4 or 5 weeks of high (8% NaCl) or low (0.3% NaCl) salt diet**. (A) Systolic blood pressure measured by tail cuff. No difference among groups at baseline. At week 4, systolic blood pressure was significantly lower in all groups compared with a high salt diet alone (P ≤ 0.05). The pooled-over-time mean of the low salt group was significantly different from the high salt group (p < 0.003) (B) Arterial blood pressure measured by implanted transmitter. *P ≤ 0.05 versus high salt diet alone. (C) Pulse pressure measured by implanted transmitter. *P ≤ 0.05 versus high salt diet alone. (D) Change in body weight. (E) Kaplan-Meier survival curves followed for 8 weeks. N = 7 to 10 rats per group (N = 3 to 8 for telemetry data). Mean ± SEM.

General model characteristics are shown in figure 1. After 4 weeks on a high salt diet, systolic BP increased 60%. In contrast, systolic BP in rats fed a low salt diet increased only 17%. Mean arterial pressure (MAP) remained relatively unchanged in low salt rats, but increased by 73% in high salt rats. Pulse pressure increased by 15% and 68%, respectively. Body weight gain was similar. Treatment with AC3174, captopril, GLP-1, or the combination of AC3174 plus captopril reduced systolic BP compared with high salt diet alone (P ≤ 0.05), but had no effect on body weight gain. Further, the development of cardiometabolic and renal disease in high salt rats had a dramatic effect on survival, with 50% mortality before week 5 and zero survival by week 7. In contrast, none of the low salt rats died during the 8-week observation period. AC3174, captopril, and AC3174 plus captopril all lengthened survival among high salt rats (P ≤ 0.05), with AC3174 plus captopril being the most beneficial. The predicted 50% survival weeks were 5.5 (high salt), 5.7 (GLP-1), 7.8 (captopril), 9.4 (AC3174), and 10.4 (AC3174 plus captopril).

**Figure 2:**
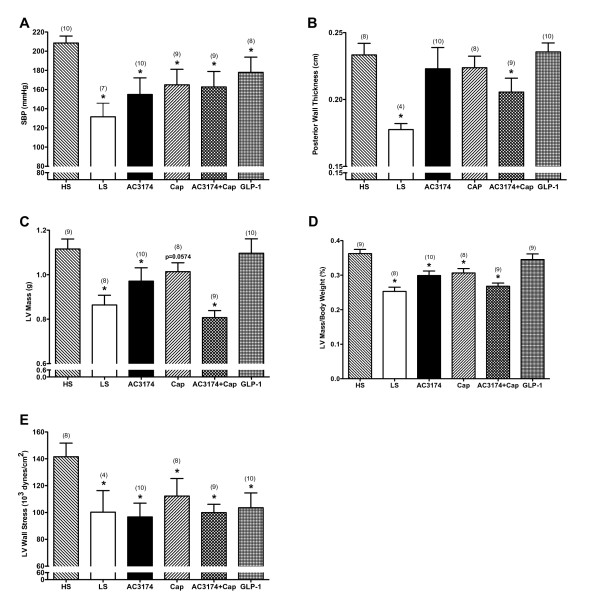
**Cardiovascular changes after 4 weeks of treatment demonstrated beneficial effects of AC3174**. (A) Systolic blood pressure. P = 0.0226 for one-way ANOVA. The pooled-over-time means of the low salt and AC3174 groups were significantly different from the high salt group (P ≤ 0.05). (B) Posterior left ventricular wall thickness. P = 0.0030 for one-way ANOVA. The pooled-over-time mean of the low salt group was significantly different from the high salt group (P ≤ 0.05). (C) Left ventricular heart mass. P = 0.0002 for one-way ANOVA. The pooled-over-time means of the low salt and AC3174 plus captopril groups were significantly different from the high salt group (P ≤ 0.05). (D) Left ventricular heart mass as a percentage of total body weight. P < 0.0001 for one-way ANOVA. The pooled-over-time means for all treatment groups, except GLP-1, were significantly different from the high salt group (P ≤ 0.05). (E) Left ventricular heart wall stress. P = 0.0512 for one-way ANOVA. The pooled-over-time means of the AC3174 and AC3174 plus captopril groups were significantly different from the high salt group (P ≤ 0.05). There were 10 rats per group at beginning of treatment. N = 3 to 10 rats per group at end of treatment actually shown. *P < 0.05 versus high salt diet alone. Mean ± SEM.

The deleterious cardiovascular effects of a high salt diet were ameliorated to different degrees by the tested treatments (figure 2). Systolic BP was significantly lower in low salt rats (132 ± 14 mmHg) than in high salt rats (209 ± 7 mmHg; P ≤ 0.05). AC3174 (155 ± 17 mmHg), captopril (165 ± 16 mmHg), GLP-1 (178 ± 14 mmHg), and AC3174 plus captopril (163 ± 16 mmHg) all significantly reduced systolic BP versus high-salt rats (P ≤ 0.05), with GLP-1 having the least effect. In concert with worsening hypertension, LV mass and ventricular wall thickness both increased compared with low salt rats. AC3174 plus captopril attenuated the effects of high salt on posterior wall thickness, LV mass, and the ratio of LV mass to total body weight (P ≤ 0.05). However, GLP-1 treatment had no effect. The ratio of LV mass to total body weight was also reduced by a low salt diet, AC3174 monotherapy, or captopril monotherapy compared with a high salt diet alone (P ≤ 0.05). All treatments reduced LV wall stress: 22% (low salt), 30% (AC3174), 20% (captopril), 28% (combination), and 27% (GLP-1) compared with high salt alone (P ≤ 0.05).

**Figure 3:**
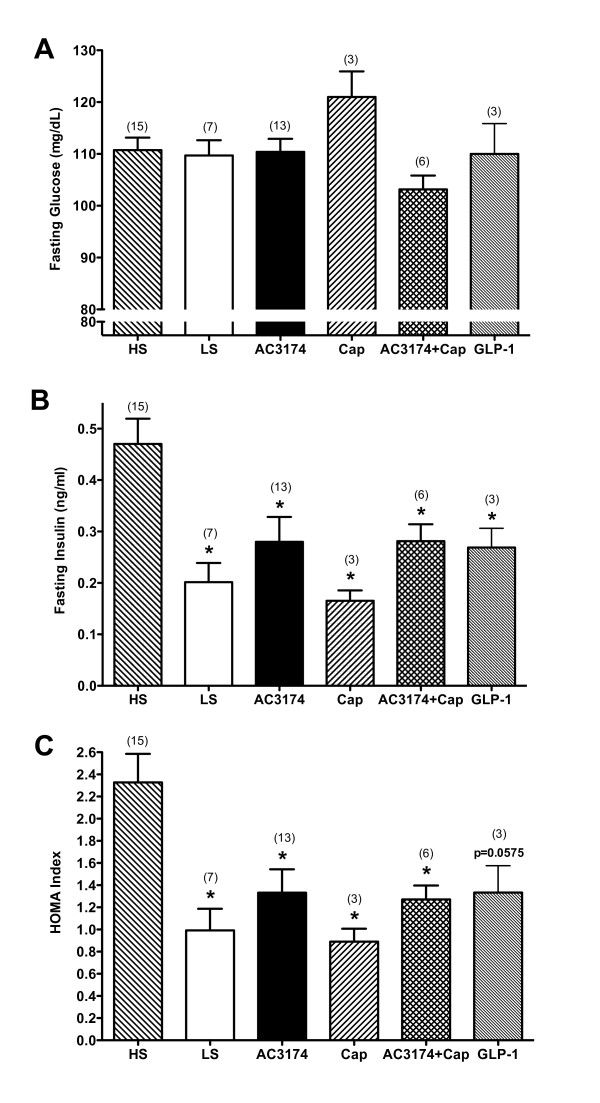
**Beneficial effects of AC3174 on fasting glycemic control after 4 weeks of treatment**. (A) Fasting serum glucose concentrations. No significant differences among groups. (B) Fasting serum insulin concentrations. P = 0.0020 for one-way ANOVA. The pooled-over-time means of the low salt, AC3174, and captopril groups were significantly different from the high salt group (P ≤ 0.05). (C) HOMA. P = 0.0014 for one-way ANOVA. The pooled-over-time means for all treatment groups except GLP-1 were significantly different from the high salt group (P ≤ 0.05).

There were no effects on fasting glucose in this normoglycemic rat model (figure 3). However, compared with low salt rats, fasting insulin and HOMA-IR were significantly increased by 2.3-fold in high salt rats (P ≤ 0.05), indicating insulin resistance. GLP-1, AC3174, captopril, or AC3174 plus captopril normalized fasting insulin and HOMA-IR (P ≤ 0.05).

**Figure 4:**
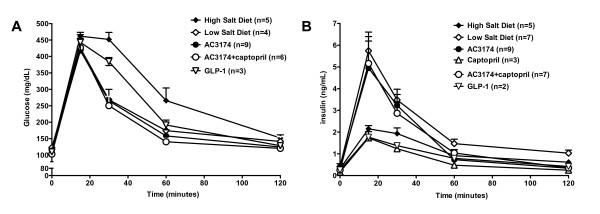
**Beneficial effects of AC3174 on postprandial glycemic control after 4 weeks of treatment**. (A) Serum glucose concentrations after an intraperitoneal bolus of glucose. No significant difference between the GLP-1 and high salt alone groups. At 30 min post-bolus, low salt, AC3174, GLP-1, and combination AC3174 plus captopril treatment significantly lowered glucose concentrations compared with high salt diet alone (P ≤ 0.05). At 60 min post-bolus, low salt, AC3174, and AC3174 plus captopril significantly lowered glucose concentrations compared with high salt diet alone (P ≤ 0.05). At 120 min post-bolus, AC3174 and AC3174 plus captopril significantly lowered glucose concentrations compared with high salt diet alone (P ≤ 0.05). No data were collected from the captopril group. (B) Serum insulin concentrations after an intraperitoneal bolus of glucose. A high salt diet lowered insulin concentrations at all post-bolus time points compared with a low salt diet (P ≤ 0.05). No significant difference between the GLP-1 and high salt groups. Captopril significantly lowered insulin concentrations at 30, 60, and 120 min post-bolus compared with a high salt diet (P ≤ 0.05). Treatment with AC3174 (alone or with captopril) significantly raised insulin concentrations at 15 and 120 min post-bolus compared with high salt diet alone (p < 0.05). *P ≤ 0.05 versus high salt diet. N = 3 to 14 rats per group. Mean ± SEM.

Postprandial glucose concentrations remained elevated in high salt DSS rats at 30 and 60 minutes post-glucose load (figure 4). This was in sharp contrast with the low salt, AC3174, or AC3174 plus captopril groups, where indistinguishable and more rapid normalization occurred. GLP-1 was less effective. Postprandial insulin had a more variable treatment response. Insulin levels in rats fed high salt alone, GLP-1, or captopril monotherapy were similarly blunted after the glucose bolus. However, in the low salt, AC3174 monotherapy, and combination groups, postprandial insulin rapidly increased to levels ~2.4-fold higher than in the high salt group by 15 minutes post-bolus and remained generally elevated until 60 minutes post-bolus.

**Figure 5:**
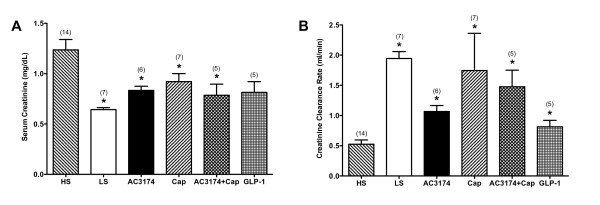
**Beneficial effects of AC3174 on renal function after 4 weeks of treatment**. (A) Serum creatinine concentrations. P = 0.0003 for one-way ANOVA. (B) Glomerular filtration rate measured by creatinine clearance. P = 0.0001 for one-way ANOVA. *P ≤ 0.05 versus high salt diet. N = 3 to 14 rats per group. Mean ± SEM.

**Figure 6:**
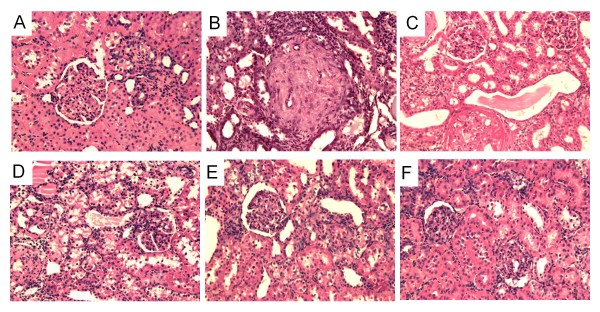
**Renal histopathology in DSS rats after 4 weeks of treatment demonstrated beneficial effects of AC3174**. (A) Low salt diet exhibiting normal-to-mild scattered sclerosis. (B) High salt diet showing diffuse severe sclerosis. (C) AC3174 treatment on a background of a high salt diet displaying scattered moderate sclerosis. (D) Captopril treatment on a background of a high salt diet showing diffuse mild sclerosis. (E) AC3174 plus captopril treatment on a background of a high salt diet showing scattered mild sclerosis. (F) GLP-1 treatment on a background of a high salt diet exhibiting scattered mild sclerosis. N = 5 to 12 rats per group.

AC3174, captopril, or the combination attenuated elevated serum creatinine and improved creatinine clearance rates compared with a high salt diet alone (P ≤ 0.05; figure 5). GLP-1 improved creatinine clearance rates. Renal morphology in low salt rats (figure 6) was comparable to normal rats *(data not shown) *with no inflammation or fibrosis and minimal sclerosis (sclerosis score 0.67 ± 0.19). In contrast, a high salt diet was associated with a significantly higher degree of diffuse moderate-to-severe sclerosis (sclerosis score 3.10 ± 0.18; P ≤ 0.05 versus low salt). AC3174 (sclerosis score 2.33 ± 0.41), captopril (sclerosis score 2.33 ± 0.29), or GLP-1 (sclerosis score 2.00 ± 0.45) each visibly attenuated renal damage to a similar extent compared with a high salt diet. However, the combination of AC3174 and captopril produced the most improvement (sclerosis score 1.50 ± 0.20; p ≤ 0.05 versus high salt; p > 0.05 versus low salt).

## Discussion

Treatment of DSS rats fed a high salt diet with the exenatide analogue AC3174, captopril, or AC3174 plus captopril markedly attenuated the development of hypertension and cardiomyopathy. The overall efficacy of AC3174 was comparable to captopril. However, the effect of AC3174 was additive with captopril in normalizing LV mass. Thus, AC3174 had cardiovascular benefits beyond those achieved via ACE inhibition alone, either by enhancing captopril effectiveness, or more likely, by an independent mechanism. One possible mechanism for the additive effects of AC3174 and captopril could be increased concentrations of circulating angiotensin(1-7) [35]. In spontaneously hypertensive rats, angiotensin(1-7) was the only component of the renin-angiotensin system that was elevated compared with normotensive rats. In addition, chronic ACE inhibition resulted in elevated angiotensin(1-7) levels in both hyper- and normotensive rats.

Overall, AC3174 monotherapy or AC3174 plus captopril was more efficacious than GLP-1. In a previously reported study, GLP-1 infusion prevented cardiac hypertrophy, reduced cardiac fibrosis, lowered MAP, and partially restored endothelial function in isolated aortic rings from high salt DSS rats [13]. In salt-sensitive obese diabetic mice, exenatide attenuated the development of hypertension and body weight gain, and increased urinary sodium excretion [20]. Additionally, in a rat model of metabolic syndrome, exenatide reversed corticosterone-induced elevations in BP independent from weight change [19]. Exenatide reduced corticosterone-induced hypertension by 86%. Finally, in a pig model of acute myocardial infarction, exenatide treatment before reperfusion improved cardiac function, reduced infarct size, and decreased myocyte apoptosis within the ischemic infarct site [36]. Taken together, these data suggest that GLP-1 receptor agonists such as exenatide and AC3174 have potential as therapeutic agents for preventing and attenuating the development of hypertension and cardiac hypertrophy.

DSS rats fed a high salt diet rapidly developed profound hypertension, LV hypertrophy, insulin resistance, and renal pathology leading to early-onset mortality from hypertension-induced stroke, with 50% mortality before week 5 and zero survival by week 7. In contrast, none of the low salt DSS rats died during the 8 week observation period. AC3174, captopril, and AC3174 plus captopril all lengthened survival among high salt DSS rats, with the combination being the most effective. Previous data have demonstrated that although acute exposure to exenatide decreases food intake in the short-term, this effect disappears after a week with chronic exenatide exposure [37]. Thus, rats treated with the exenatide analogue AC3174 were exposed to equivalent salt content from food intake as other treatment groups. In contrast, GLP-1 did not improve survival. This resulted in fewer GLP-1 animals being available for end-of-study analyses.

DSS rats fed a high salt diet develop cardiac dysfunction leading to failure characterized by LV hypertrophy, increased LV wall stress, and LV fibrosis [13,26,28]. DSS rats fed a high salt diet from 7 weeks of age develop hypertension antecedent to compensatory LV hypertrophy with LV relaxation abnormalities by 13 weeks of age [26]. By 17 weeks of age, there was further progression of LV hypertrophy combined with fibrosis and myocardial stiffening. Overt diastolic heart failure, increased LV filling pressure, and pulmonary congestion occurred by approximately 20 weeks of age; followed shortly by death [26,28].

There is a strong correlation between insulin-resistance and hypertension in humans [38-40]. Insulin resistance has been linked to cardiac hypertrophy and fibrosis, endothelial dysfunction, and renal glomerulosclerosis. Hyperinsulinemia may contribute to the development of hypertension by promoting endothelial dysfunction and stimulating renal tubule reabsorption of sodium [39]. Conversely, increasing insulin sensitivity is associated with increased cardioprotection, normalized endothelial function, and reduced MAP [13,25,40].

The DSS rat model of hypertension exhibits insulin-resistance exacerbated by salt intake and age [40]. One mechanism by which high salt might precipitate insulin resistance is through its ability to enhance an oxidative stress-induced inflammatory response that disrupts the insulin signaling pathway [41]. Fujii et al. [25] reported that fatty acid oxidation and insulin-stimulated glucose uptake were impaired in high salt DSS rats with cardiac hypertrophy. Further, glucose uptake was maximally stimulated under basal conditions in the hypertrophied heart, with no residual capacity to increase glucose uptake in response to insulin administration. The new findings reported here are that treatment of high salt DSS rats with AC3174, captopril, or the combination reduced fasting insulin and insulin resistance with no effect on fasting glucose, similar to GLP-1. Chronic treatment with exenatide or GLP-1 is associated with increased insulin sensitivity in preclinical models [17,42]. Further, these changes were at least as great as the change associated with metformin, TZDs, SFUs, or insulin therapy. Given the correlation between insulin resistance, hyperinsulinemia, and hypertension leading to more serious cardiovascular disease [39], these data lend further support for the potential of AC3174 therapy in this setting. After a glucose load (postprandial state), ambient glucose concentrations remained elevated for an extended period of time in high salt DSS rats and to a slightly lesser extent in GLP-1 treated high salt rats. This was in sharp contrast to all other treatment groups where more rapid normalization of glucose levels occurred. The insulin response to a glucose load was muted in high salt rats receiving vehicle or captopril. In contrast, the insulin response in high salt rats treated with AC3174 monotherapy or AC3174 plus captopril was 3-fold more vigorous within 15 minutes post-glucose load. These data point to the ability of a GLP-1 receptor agonist to independently stimulate an insulinotropic response to glucose loading in the context of advanced cardiac and renal disease.

The RAAS plays a significant role in the emergence of LV dysfunction by instigating myocardial fibrosis, volume overload, vasoconstriction, and cardiovascular tissue inflammation [6]. After 7 weeks on a high salt diet, DSS rat kidneys are characterized by decreased function, increased proteinuria, glomerulosclerosis, increased adrenomedullin and atrial natriuretic peptide concentrations compared with salt-resistant rats [30]. DSS rats on a high salt diet develop a renal pathology resembling that observed in patients with diabetic nephropathy and hypertensive-induced end stage renal disease [13,30,43]. Supporting these findings is the demonstrated ability of ACE inhibitors, ARBs, and aldosterone antagonists to delay renal disease progression in this model [28,43]. Captopril administration to male high salt DSS rats prevented a further increase in systolic BP, but had no effect on plasma sodium concentration, plasma osmolality, or hematocrit [44]. Long-term captopril infusion slowed the expected rise in systolic BP, increased urinary protein excretion, and slowed renal glomerular sclerosis [43].

Under the conditions used in the study reported here, AC3174, captopril, or the combination improved glomerular filtration rate more than GLP-1 in DSS rats fed a high salt diet. Yu et al. [13] reported that intravenous infusion of GLP-1 into DSS rats fed a high salt diet for 2 weeks attenuated the development of hypertension, renal proteinuria, and renal albuminuria. GLP-1 also reduced glomerulosclerosis, renal tubule necrosis, and the degree of renal interstitial fibrosis in the outer medulla. Taken together, these data suggest that GLP-1 receptor agonists can promote renal salt and water excretion by inhibiting tubular reabsorption of sodium, a mechanism contributing to the antihypertensive effects in this model.

In the kidneys of high salt DSS rats, more than 75% of the area of the glomerular capillaries can be filled with matrix material, indicating a high degree of glomerular injury [13]. This pathology is accompanied by a marked necrosis of renal tubules and formation of protein casts in the outer medulla. In one study, GLP-1 normalized renal morphology to a degree comparable to kidneys in low salt DSS rats [13]. As reported here, the morphology of high salt DSS rat kidneys showed a high degree of sclerosis that was improved comparably by AC3174 or captopril monotherapy. Notably, combination treatment with AC3174 and captopril further improved renal sclerosis and this effect was better than that induced by GLP-1 monotherapy. Since the anti-hypertensive effects were comparable among AC3174, captopril, and AC3174 plus captopril, the renal histopathological improvements from AC3174 plus captopril suggest an additional direct effect of AC3174 on the heart and kidney combined with the possibility of indirect effects from lowered BP.

## Conclusions

DSS rats fed a high salt diet rapidly developed profound hypertension, cardiac hypertrophy, insulin resistance, and renal pathology leading to early-onset mortality. AC3174, captopril, and AC3174 plus captopril all lengthened survival, in contrast to GLP-1. AC3174 had anti-hypertensive, insulin-sensitizing, and renoprotective effects. The overall efficacy of AC3174 was comparable to that of captopril and better than GLP-1. However, the effects of AC3174 were additive with captopril in reducing LV mass and improving renal morphology, and AC3174 independently stimulated an insulinotropic response to glucose loading in the context of advanced cardiac and renal disease. These data suggest further clinical investigations of GLP-1 receptor agonists in the treatment of cardiorenal syndrome and hypertension are warranted.

## Abbreviations

GLP-1: glucagon-like peptide-1; DSS: Dahl salt-sensitive; LS: low salt; HS: high salt; LV: left ventricular; SBP: systolic blood pressure; DBP: diastolic blood pressure; HOMA-IR: homeostasis model assessment-insulin resistance; ACE: angiotensin-converting enzyme; RAAS: renin-angiotensin aldosterone system.

## Competing interests

All authors were employees and held stock options in Amylin Pharmaceuticals, Inc. at the time these experiments were performed.

## Authors' contributions

QL helped conceive of the study, and participated in its design, analysis, interpretation, coordination and helped draft the manuscript. LA participated in the design and coordination of the study and helped carry out the in vivo, cell biology and histological studies. AB participated in the design and coordination of the study and helped carry out the in vivo studies. RF participated in the design and coordination of the study and helped carry out the in vivo and molecular genetic studies. ADB helped conceive of the study, and participated in its design, analysis and interpretation. DGP helped conceive of the study, participated in its design, analysis, interpretation and helped draft the manuscript. All authors read and approved the final manuscript.

## References

[B1] Thom T Haase N Rosamond W Howard VJ Rumsfeld J Manolio T Zheng Z Flegal K O'Donnell C Kittner S Lloyd-Jones D Goff DC Hong Y Members of the Statistics Committee and Stroke Statistics Subcommittee Adams R Friday G Furie K Gorelick P Kissela B Marler J Meigs J Roger V Sidney S Sorlie P Steinberger J Wasserthiel-Smoller S Wilson M Wolf P Heart disease and stroke statistics - 2006 update: a report from the American Heart Association Statistics Committee and Stroke Statistics Subcommittee Circulation 2006 113 e85 e151 10.1161/CIRCULATIONAHA.105.171600 16407573

[B2] Chobanian AV the National Committee The Seventh Report of the Joint National Committee on Prevention, Detection, Evaluation, and Treatment of High Blood Pressure (JNC 7) 2004 Bethesda, MD: National Institutes of Health, U.S. Department of Health and Human Services NIH Publication No. 04-5230

[B3] Ong KL Cheung BMY Man YB Lau CP Lam KSL Prevalence, awareness treatment, and control of hypertension among United States adults 1999-2004 Hypertension 2007 49 69 75 10.1161/01.HYP.0000252676.46043.18 17159087

[B4] Lalande S Johnson BD Diastolic dysfunction: a link between hypertension and heart failure Drugs Today 2008 44 503 513 10.1358/dot.2008.44.7.1221662 18806901 PMC2713868

[B5] Verma A Solomon SD Diastolic dysfunction as a link between hypertension and heart failure Med Clin N Am 2009 93 647 664 10.1016/j.mcna.2009.02.013 19427497

[B6] Sciarretta S Paneni F Palano F Chin D Tocci G Rubattu S Volpe M Role of the renin-angiotensin-aldosterone system and inflammatory processes in the development and progression of diastolic dysfunction Clin Sci 2009 116 467 477 10.1042/CS20080390 19200056

[B7] Sander GE Giles TD Diabetes mellitus and heart failure Amer Heart Hosp J 2003 1 273 280 10.1111/j.1541-9215.2003.02085.x 15815121

[B8] Reboldi G Gentile G Angeli F Verdecchia P Choice of ACE inhibitor combinations in hypertensive patients with type 2 diabetes: update after recent clinical trials Vasc Hlth Risk Manag 2009 5 411 427 10.2147/vhrm.s4235 PMC2686259 19475778

[B9] Drucker DJ The role of gut hormones in glucose homeostasis J Clin Invest 2007 117 24 32 10.1172/JCI30076 17200703 PMC1716213

[B10] Nikolaidis LA Sunil Mankad S Sokos GG Miske G Shah A Elahi D Shannon RP Effects of glucagon-like peptide-1 in patients with acute myocardial infarction and left ventricular dysfunction after successful reperfusion Circulation 2004 109 962 965 10.1161/01.CIR.0000120505.91348.58 14981009

[B11] Kavianipour M Ehlers MR Malmberg K Ronquist G Ryden L Gutniak Wikstrom G Glucagon-like peptide-1 (7-36) amide prevents the accumulation of pyruvate and lactate in the ischemic and non-ischemic porcine myocardium Peptides 2003 24 569 578 10.1016/S0196-9781(03)00108-6 12860201

[B12] Nikolaidis LA Elahi D Hentosz t Doverspike A Huerbin R Zourelias L Stolarski C Shen YT Shannon RP Recombinant glucagon-like peptide-1 increases myocardial glucose uptake and improves left ventricular performance in conscious dogs with pacing-induced dilated cardiomyopathy Circulation 2004 110 955 961 10.1161/01.CIR.0000139339.85840.DD 15313949

[B13] Yu M Moreno C Hoagland KM Dahly A Ditter K Mistry M Roman RJ Antihypertensive effects of glucagon-like peptide-1 in Dahl salt-sensitive rats J Hypertension 2003 21 1125 1135 10.1097/00004872-200306000-00012 12777949

[B14] Nielsen LL Young AA Parkes DG Pharmacology of exenatide (synthetic exendin 4): a potential therapeutic for improved glycemic control of type 2 diabetes Regul Pept 2004 117 77 88 10.1016/j.regpep.2003.10.028 14700743

[B15] Nielsen LL Okerson T Holcombe J Hoogwerf B Effects of exenatide on diabetes, obesity, cardiovascular risk factors, and hepatic biomarkers in patients with type 2 diabetes J Diab Sci Tech 2008 2 255 260 10.1177/193229680800200214 PMC2771486 19885351

[B16] Malone J Trautmann M Wilhelm K Taylor K Kendall DM Exenatide once weekly for the treatment of type 2 diabetes Expert Opin Investig Drugs 2009 18 359 367 10.1517/13543780902766802 19243286

[B17] Gedulin BR Nikoulina SE Smith PA Gedulin G Nielsen LL Baron AD Parkes D Young AA Exenatide (exendin-4) improves insulin sensitivity and β-cell mass in insulin-resistant obese *fa/fa *Zucker rats independent of glycemia and body weight Endocrinology 2005 146 2069 2076 10.1210/en.2004-1349 15618356

[B18] Gardiner SM March JE Kemp PA Bennett T Mesenteric vasoconstriction and hindquarters vasodilatation accompany the pressor actions of exendin-4 in conscious rats J Pharmacol Exp Ther 2006 316 852 859 10.1124/jpet.105.093104 16221740

[B19] Laguero KD Stonehouse AH Guss S Landry J Vu C Parkes DG Exenatide improves hypertension in a rat model of the metabolic syndrome Metabolic Syndr Rel Disorders 2009 7 327 334 10.1089/met.2008.0095 19320558

[B20] Hirata K Kume S Araki S Sakaguchi M Chin-Kanasaki M Isshiki K Sugimoto T Nishiyama A Koya D Haneda M Kashiwagi A Uzu T Exendin-4 has an anti-hypertensive effect in salt-sensitive mice model Biochem Biophys Res Commun 2009 380 44 49 10.1016/j.bbrc.2009.01.003 19150338

[B21] Gutzwiller J Tschopp S Bock A Zehnder CE Huber AR Kreyenbuehl M Gutmann H Drewe J Henzen C Goeke B Beglinger C Glucagon-like peptide 1 induces natriuresis in healthy subjects and in insulin-resistant obese men J Clin Endocrinol Metab 2004 89 3055 3061 10.1210/jc.2003-031403 15181098

[B22] Blonde L Klein EJ Han J Zhang B Mac SM Poon TH Taylor KL Trautmann ME Kim DD Kendall DM Interim analysis of the effects of exenatide treatment on A1C, weight and cardiovascular risk factors over 82 weeks in 314 overweight patients with type 2 diabetes Diabetes Obes Metab 2006 8 436 447 10.1111/j.1463-1326.2006.00602.x 16776751

[B23] Moretto TJ Milton DR Ridge TD MacConell LA Okerson T Wolka AM Brodows RG Efficacy and tolerability of exenatide monotherapy over 24 weeks in antidiabetic drug-naive patients with type 2 diabetes: a randomized, double-blind, placebo-controlled, parallel-group study Clin Ther 2008 30 1448 1460 10.1016/j.clinthera.2008.08.006 18803987

[B24] Okerson T Yan P Stonehouse A Brodows R Effects of exenatide on systolic blood pressure in subjects with type 2 diabetes Amer J Hypertens 2009 23 334 339 10.1038/ajh.2009.245 20019672

[B25] Fujii N Nozawa T Igawa A Kato B Igarashi N Nonomura M Asanoi H Tazawa S Inoue M Inoue H Saturated glucose uptake capacity and impaired fatty acid oxidation in hypertensive hearts before development of heart failure Am J Physiol Heart Circ Physiol 2004 287 H760 766 10.1152/ajpheart.00734.2003 15031123

[B26] Yoshida J Yamamoto K Mano T Sakata Y Nishikawa N Nishio M Ohtani T Miwa T Hori M Masuyama T AT1 receptor blocker added to ACE inhibitor provides benefits at advanced stage of hypertensive diastolic heart failure Hypertens 2004 43 686 691 10.1161/01.HYP.0000118017.02160.fa 14757777

[B27] Sakata Y Yamamotoa K Manoa T Nishikawaa N Yoshida J Miwa T Hori M Masuyama T Temocapril prevents transition to diastolic heart failure in rats even if initiated after appearance of LV hypertrophy and diastolic dysfunction Cardiovasc Res 2003 57 757 765 10.1016/S0008-6363(02)00722-8 12618237

[B28] Pinto YM Paul M Ganten D Lessons from rat models of hypertension: from Goldblatt to genetic engineering Cardiovasc Res 1998 39 77 88 10.1016/S0008-6363(98)00077-7 9764191

[B29] Reaven GM Twersky J Chang H Abnormalities of carbohydrate and lipid metabolism in Dahl rats Hypertension 1991 18 630 635 1937666 10.1161/01.hyp.18.5.630

[B30] Nishikimi T Akimotob K Wanga X Moria Y Tadokoroa K Ishikawaa Y Shimokawac H Ono H Matsuoka H Fasudil, a Rho-kinase inhibitor, attenuates glomerulosclerosis in Dahl salt-sensitive rats J Hypertens 2004 22 1787 1796 10.1097/00004872-200409000-00024 15311108

[B31] Maric C Sandberg K Hinojosa-Laborde C Glomerulosclerosis and tubulointerstitial fibrosis are attenuated with 17β-estradiol in the aging Dahl salt sensitive rat J Am Soc Nephrol 2004 15 1546 1556 10.1097/01.ASN.0000128219.65330.EA 15153565

[B32] Hargrove DM Kendall ES Reynolds JM Lwin AN Herich JP Smith PA Gedulin BR Flanagan SD Jodka CM Hoyt JA McCowen KM Parkes DG Anderson CM Biological activity of AC3174, a peptide analog of exendin-4 Reg Pept 2007 141 113 119 10.1016/j.regpep.2006.12.021 17292977

[B33] Dahl LK Heine M Tassinari L Effects of chronic excess salt ingestion: evidence that genetic factors play an important role in susceptibility to experimental hypertension J Exp Med 1962 115 1173 1190 10.1084/jem.115.6.1173 13883089 PMC2137393

[B34] Parkes D Jodka C Smith P Nayak S Rinehart L Gingerich R Chen K Young A Pharmacokinetic actions of exendin-4 in the rat: comparison with glucagon-like peptide-1 Drug Dev Res 2001 53 260 267 10.1002/ddr.1195

[B35] Kohara K Brosnihan B Ferrario CM Angiotensin(1-7) in the spontaneously hypertensive rat Peptides 1993 14 883 891 10.1016/0196-9781(93)90063-M 8284265

[B36] Timmers L Henriques JPS de Kleijn DPV DeVries JH Kemperman H Steendijk P Verlaan CWJ Kerver M Piek JJ Doevendans PA Pasterkamp G Hoefer IE Exenatide reduces infarct size and improves cardiac function in a porcine model of ischemia and reperfusion injury J Am Coll Cardiol 2009 53 501 510 10.1016/j.jacc.2008.10.033 19195607

[B37] Mack CM Moore CX Jodka CM Bhasvsar S Wilson JK Hoyt JA Roan JL Vu C Laugero KD Parkes DG Young AA Antiobesity action of peripheral exenatide (exendin-4) in rodents: effects on food intake, body weight, metabolic status and side-effect measures Int J Obesity 2006 30 1332 1340 10.1038/sj.ijo.0803284 16534527

[B38] van Heerebeek L Somsen A Paulus WJ The failing diabetic heart: focus on diastolic left ventricular dysfunction Curr Diab Rep 2009 9 79 86 10.1007/s11892-009-0014-9 19192429

[B39] Kashyap SR DeFronzo RA The insulin resistance syndrome: physiological considerations Diab Vasc Dis Res 2007 4 13 19 10.3132/dvdr.2007.001 17469039

[B40] Shehata MF Genetic and dietary salt contributors to insulin resistance in Dahl salt-sensitive (S) rats Cardiovasc Diabetol 2008 7 7 10.1186/1475-2840-7-7 18397529 PMC2365939

[B41] Shehata MF Important genetic checkpoints for insulin resistance in salt-sensitive (S) Dahl rats Cardiovasc Diabetol 2008 7 19 10.1186/1475-2840-7-19 18570670 PMC2459151

[B42] Young AA Hansen B, Shafir E Glucagon-like peptide-1, exendin and insulin sensitivity Insulin resistance and insulin resistance syndrome 2002 Chapter 14 New York: Taylor and Francis 235 262

[B43] Ideishi M Miura S Sakai T Maeda H Kinoshita A Sasaguri M Jimi S Arakawa K Comparative effects of an angiotensin-converting enzyme inhibitor and an angiotensin II antagonist in Dahl rats Blood Pressure 1994 3 Suppl 5 99 104 7889212

[B44] Crofton JT Ota M Share L Role of vasopressin, the renin-angiotensin system and sex in Dahl salt-sensitive hypertension J Hypertens 1993 11 1031 1038 10.1097/00004872-199310000-00005 8258666

